# The World Health Organization Safe Childbirth Checklist on Essential Birth Practices and Perinatal Mortality: A Meta-Analysis

**DOI:** 10.1001/jamanetworkopen.2025.58269

**Published:** 2026-02-26

**Authors:** Lennart Christian Kaplan, Megan Marx Delaney, Pia Roddewig, Shambhavi Singh, Rose L. Molina, Farah Diba, Danielle E. Tuller, Lauren Bobanski, Ashfa Hashmi, Marthoenis Marthoenis, Katharina Richert, Ichsan Ichsan, Vinay Pratap Singh, Muhsin Muhsin, Vishwajeet Kumar, Hizir Sofyan, Sebastian Vollmer, Katherine E. A. Semrau

**Affiliations:** 1 Georg-August-University of Göttingen, Göttingen, Germany; 2 Ariadne Labs, Brigham and Women’s Hospital, Harvard T.H. Chan School of Public Health, Boston, Massachusetts; 3 Department of Economics & Centre for Modern Indian Studies, University of Goettingen, Göttingen, Germany; 4 Community Empowerment Lab, Lucknow, India; 5 Beth Israel Deaconess Medical Center, Boston, Massachusetts; 6 Community Health Nursing Department, Faculty of Nursing, Universitas Syiah Kuala, Banda Aceh, Indonesia; 7 World Health Organization Country Office, National Institutes of Health, Chak Shahzad, Islamabad, Pakistan; 8 Department of Psychiatry and Mental Health Nursing. Universitas Syiah Kuala, Banda Aceh, Indonesia; 9 Center for Evaluation and Development, Mannheim, Germany; 10 Medical Research Unit, School of Medicine, Universitas Syiah Kuala, Banda Aceh, Indonesia; 11 Department of Microbiology, School of Medicine, Universitas Syiah Kuala, Banda Aceh, Indonesia; 12 Tsunami and Disaster Mitigation Research Center, Universitas Syiah Kuala, Banda Aceh, Indonesia; 13 Community Empowerment Lab, Lucknow, India; 14 Faculty of Medicine, Universitas Syiah Kuala, Banda Aceh, Indonesia; 15 Community Empowerment Lab, Lucknow, India; 16 Statistics Department, Universitas Syiah Kuala, Banda Aceh, Indonesia; 17 Centre for Modern Indian Studies (CeMIS), Georg-August-University of Göttingen, Göttingen, Germany; 18 Division of Global Health Equity, Brigham and Women’s Hospital, Boston, Massachusetts; 19 Department of Medicine, Harvard Medical School, Boston, Massachusetts

## Abstract

**Importance:**

The World Health Organization (WHO) Safe Childbirth Checklist (SCC) has been adapted and implemented in at least 35 countries. Consistently, the SCC has shown increased adherence to practices, but there are mixed results regarding its association with health outcomes in different settings.

**Objective:**

To examine the association of SCC implementation with mortality, accounting for variations in evidence-based practices (EBP) adherence.

**Design, Setting, and Participants:**

In this meta-analysis, data were pooled from 3 cluster randomized trials of the SCC (January 1, 2014, to December 31, 2017). Intention to treat (ITT) and a complier average causal effect analysis (CACE) on EBPs and perinatal mortality were estimated via a generalized linear model. The primary facilities were in Uttar Pradesh, India; basic emergency obstetric facilities were in Aceh, Indonesia; and primary and secondary health centers were in Khyber Pakhtunkhwa, Pakistan.

**Interventions:**

In India, the 8-month SCC intervention involved facility engagement, a launch event, and 8 months of tapered coaching. In Indonesia, the 6-month SCC intervention included 11 coaching visits. In Pakistan, the 12-month SCC intervention included light touch external monitoring, skills training, and supplies assessment.

**Main Outcomes and Measures:**

Primary outcomes were stillbirth and perinatal and early neonatal mortality. Secondary outcomes were adherence to 15 EBPs, facility supply availability, and safety culture perceptions.

**Results:**

Pooled data included 169 511 births, supply assessments from 163 facilities, and 6298 observed deliveries for EBPs and health workers’ perceptions on safety culture. Mortality did not differ in the full sample; however, during months when EBP observations were conducted, stillbirth rates in the intervention facilities were lower by 9.8 per 1000 births (95% CI, −18.5 to −1.1; *P* = .03; q = .05) in the ITT analysis and 14.5 per 1000 births (95% CI, −27.2 to −1.7; *P* = .03; q = .05) in the CACE analysis compared with control facilities. EBP adherence was higher by 3.6 practices (95% CI, 3.3 to 4.1; *P* < .001; q = .001) in the ITT analysis and 6.0 practices (95% CI, 5.3 to 6.8; *P* < .001; q = .001) in the CACE analysis in intervention facilities.

**Conclusions and Relevance:**

In this meta-analysis, SCC use in lower-middle-income settings was associated with increased EBP adherence and lower rates of stillbirths when EBPs were directly observed. Further research is needed to identify additional factors to optimize SCC’s potential impact on maternal and newborn safety outcomes.

## Key Points

**Question:**

Is the World Health Organization (WHO) Safe Childbirth Checklist associated with evidence-based practices (EBPs) and mortality?

**Findings:**

In this meta-analysis, encompassing more than 6000 standardized observations and health data from birth registries on more than 160 000 mother-newborn pairs across India, Indonesia, and Pakistan, adherence to EBPs increased by 24 percentage points with the use of the WHO Safe Childbirth Checklist. Although no difference in mortality was detected in the full sample, stillbirth rates were 9.76 per 1000 births lower when checklist use was observed.

**Meaning:**

The WHO Safe Childbirth Checklist was associated with increased application of EBPs and lower stillbirth rates when implemented in settings that enable compliance with the checklist.

## Teaser

This meta-analysis uses data from cluster randomized trials in India, Indonesia, and Pakistan to examine the outcomes of the implementation of the World Health Organization Safe Childbirth Checklist.

## Introduction

Global maternal and newborn mortality have decreased since 2000, yet approximately 295 000 maternal deaths, 2 million stillbirths, and 2.5 million neonatal deaths occur annually.^1,2^ To enhance survival, improvements in quality of care and health system strengthening are required.^3^ To address quality of care around facility-based childbirth, the World Health Organization (WHO) Safe Childbirth Checklist (SCC) was developed in 2009 to guide birth attendants through 28 evidence-based practices (EBPs) across 4 critical pause points: on admission, just before pushing or cesarean birth, 1 hour after birth, and at discharge.^4,5^

The SCC has been adapted and implemented in at least 35 high-, middle-, and low-income countries and evaluated through small implementation studies, large government programs, and large-scale randomized clinical trials.^6,7,8,9,10,11^ These consistently show improved practice adherence, with mixed results on health outcomes.

To understand the outcomes of SCC implementation across different contexts, this meta-analysis pools data from 3 cluster randomized trials in India, Indonesia, and Pakistan. We provide evidence on the association between SCC implementation and EBP adherence as well as in-facility perinatal mortality (stillbirth and early neonatal death) as primary outcomes and essential birth supplies and birth attendant perceptions of facility safety culture as secondary outcomes.^12^ We further assess heterogeneous treatment effects contingent on baseline mortality, availability of essential supplies, annual birth volume, and birth attendant years of experience.

## Methods

We focused on the 3 existing cluster randomized trials that compare the intervention to a standard of care control group without the SCC (in contrast to other randomized clinical trials that used the SCC in the control arm).^9^ At the time these trials were conducted, all 3 countries were classified by the World Bank as lower-middle-income countries. All 3 interventions implemented a locally adapted SCC, a launch event, and facility-based coaching or monitoring structures. Birth attendant types varied across studies and include obstetricians, midwives, nurse midwives, and other clinicians providing birth-related care. Descriptions of included studies have been published elsewhere.^13,14,15^ A brief description of key components relevant to data pooling can be found in the eMethods in Supplement 1. We used deidentified datasets provided by the study principal investigators; no additional ethical review was completed for this meta-analysis. All 3 primary studies^13,14,15^ had extensive institutional review board review and consent processes described previously. The primary study protocols were approved by ethics review boards at investigators’ institutions and review boards in India, Indonesia, and Pakistan.

### Outcomes

Given the original study teams’ involvement, we had access to the primary data and examined data consistency by comparison to the primary studies. No inconsistencies were found. Our meta-analysis followed the Preferred Reporting Items for Systematic Reviews and Meta-Analyses of Individual Participant Data (PRISMA-IPD) guideline.

Exchanging protocols and surveys before baseline data collection enabled harmonization and pooled analysis of 4 groups of outcomes. We select EBPs and mortality as our primary outcomes and consider supplies and perceptions as secondary outcomes.^13,14,15^ The theory of change suggests that the mortality reducing effect of the SCC runs through the adherence to EBPs, which we could only credibly measure via clinical observations. Although all the original studies considered EBPs and mortality as outcomes in their original study protocols, supplies and perceptions were only explicitly mentioned in a subset of protocols.^13,14,15^

First, we standardized the EBP observation data available from each study. Long-term presence of observation teams reduced bias (usually a minimum of 6 days per week). Observers recorded 5785 births in India, 353 in Indonesia, and 246 in Pakistan. In India, EBPs were collected from 30 facilities from 1 region of the study’s 5 regions at 3 points in time with different coaching intensity (which we consider in a robustness test). Observations were conducted in 7 hospitals in Pakistan and 17 facilities in Indonesia based on purposefully sampling facilities with sufficient case numbers. The number of observed births per SCC pause point varies because changes in observation team shifts and referrals prevented us from observing the full birthing process for all births. Observers also tracked SCC use (eg, whether birth attendants picked up the tool or used it during one of the pause points).

Second, we collected data on mortality across settings, including perinatal deaths, stillbirths, and newborn deaths. In India, the study included 157 145 mother-newborn pairs from facility records. In Indonesia and Pakistan, the study included 5780 and 2839 mother-newborn pairs from facility registries, respectively.

Third, because effective SCC use requires the availability of essential birth supplies, the teams compiled information on the availability of 22 supplies at endline. Although data collectors checked directly for the availability of supplies in India and Pakistan, the assessment in Indonesia was based on interviews with head midwives and facility leadership.

Fourth, we assessed birth attendants’ perceptions on various safety culture items based on Likert scale questions (eg, availability of services, information, and resources). Across settings, we were able to harmonize 3 key measures on performance of practices, resource access, and a general safety assessment (items are detailed in eMethods in Supplement 1). We rescaled Likert scales (ranges, 1-4 and 1-6) as continuous 0- to 1-point scales by dividing the scores by the top value.

### Other Facility Measures

Other than the outcome measures after introduction of the intervention, the 3 trials captured information on baseline characteristics of annual birth volume, mortality, birth attendant experience, and availability of 16 supplies at baseline. Those measures were considered in an explorative analysis of heterogeneous treatment effects.

### Statistical Analysis

Our intention-to-treat (ITT) estimates regress each of the 15 binary outcomes of adherence to individual EBPs separately on a binary indicator of treatment assignment. We use a generalized linear model estimation via a logit-link function, assuming a binomial distribution, because outcomes are bounded between 0 and 1. ITT estimates in the main results consider the initial random treatment assignment at the facility level, irrespective of health workers using the SCC. Furthermore, we conducted a post hoc complier analysis aiming to estimate the average treatment effect on the individuals who adhered to the SCC (picked up and/or actively used the tool during birth), called the complier average causal effect (CACE).^16^

The CACE estimator follows a 2-step approach, which is analogous to an instrumental variable approach and uses random assignment as the instrument for adherence. In the first stage, we regress adherence to the treatment assignment. For the analysis of EBPs, adherence is measured at individual birth; for example, a checklist was considered adhered to when the observers noted that the attending staff had filled out the SCC or referred to (observed) the tool itself during the birth process (India, 60%; Indonesia, 36%; and Pakistan, 54%). Specifically, we regress this binary adherence measure on the treatment assignment using a generalized linear model estimation via a logit-link function, assuming a binomial distribution.

For the analysis of mortality outcomes, we do not have a birth-specific measure of adherence for all births because those are based on facility records. We measure adherence as the fraction of births with observed SSC use among all births. Because this adherence measure is continuous, we apply a linear probability model in the first stage.

In the second stage, EBPs (birth level) and mortality (facility level) are regressed on predicted compliance, relying analogously to the ITT analysis on a generalized linear model to account for the binary outcomes. The second stage effect can then be interpreted as the weighted effect for those individuals who adhered to the intervention in the treatment and control arms and is thus by definition larger than the ITT effect. Clustered SEs account for intracluster correlation at the facility level. For the CACE, we bootstrap SEs in the second stage.

We also estimate heterogeneous treatment effects of the SCC on (1) the number of total EBPs (range, 0-15) and (2) mortality rates at the facility level contingent on baseline covariates. For this purpose, we run ordinary least squares estimations, which consider interactions between the intervention and facility-level baseline characteristics (for more detail see eTables 5 and 6 in Supplement 1) for an ITT analysis. Because the heterogeneity analysis reduces degrees of freedom and statistical power, we relied for the mortality rates on the full sample (instead of the observation sample alone).

Given the multiple hypotheses tested on the same data, we also adjust for the false discovery rate (inflated type I error).^17^ For this purpose, we report q-values to indicate whether associations would be statistically significant based on a procedure implemented by Anderson in Stata.^18^ According to the general standard in the literature, our preferred significance level across estimations is set to α = .05 for each research question, with statistical significance assessed using 2-sided tests. Analyses were performed via Stata, version 17.0 (StataCorp).^19^

## Results

### Baseline Characteristics

The baseline characteristics of the trial’s facilities are described in Table 1. Mean (SD) annual birth volumes ranged from 380 (738) (Indonesia) to 1595 (524) births (India) per facility. The sample included a fair level of heterogeneity regarding baseline mortality rates. In Pakistan, in-facility mortality rates were lowest. There were no in-facility maternal deaths recorded in Pakistani facilities, and it was a rare outcome in India with 3 cases; more cases occurred in Indonesia (32 total deaths). Stillbirths were common across settings (16.1 per 1000 births). Of note, these rates did not represent the country-level mortality rates because the trials included only in-facility births. Birth attendants had comparable mean (SD) years of experience, ranging from 9.35 (5.36) to 13.84 (8.70) years across all 3 settings. Of the 16 essential birth supplies, facilities in India had a mean (SD) of 12.45 (1.75) available, whereas supply levels were higher in Indonesia (mean [SD], 14.88 [0.66]) and lower in Pakistan (mean [SD], 12.27 [2.15]). No difference in baseline characteristics between the intervention and control facilities was detected (eTable 2 in Supplement 1).

**Table 1.** Facility-Level Baseline Characteristics of the Full Sample^a^

Characteristic	India		Indonesia		Pakistan	
	Facilities, No.	Mean (SD)	Facilities, No.	Mean (SD)	Facilities, No.	Mean (SD)
Annual birth volume	120	1595.08 (523.47)	32	380.19 (737.61)	11	666.36 (1231.55)
Perinatal (in-facility) death rate per 1000	109	18.18 (9.61)	32	14.28 (22.41)	11	4.75 (6.18)
Early neonatal (in-facility) death rate per 1000	109	0.13 (0.43)	32	2.24 (5.63)	11	0
Stillbirth (in-facility) rate per 1000	106	18.56 (9.24)	32	12.04 (22.02)	11	4.75 (6.18)
Maternal (in-facility) death rate per 100 000	109	0	31	125.76 (396.43)	11	0
Birth attendant experience, y	120	9.97 (4.78)	32	9.35 (5.36)	12	13.84 (8.70)
No. of supplies of 16 supplies	120	12.45 (1.75)	32	14.88 (0.66)	11	12.27 (2.15)

^a^ Observation numbers refer to the facility level for the facilities considered in the intention-to-treat analysis. The baseline information could not be obtained for all facilities across countries (120 in India, 32 in Indonesia, and 12 in Pakistan). The baseline characteristics of the observation sample are provided in eTable 1 in Supplement 1. Supply count refers to a facility-level count of a maximum of 16 available supplies (for a list see the eMethods in Supplement 1).

Table 2 presents EBP adherence in control facilities (because we did not conduct observations at baseline). Descriptive statistics were structured along the 4 pause points: at admission (P1), shortly before birth (P2), 1 minute after birth (P3), and within 1 hour after birth (P4). Adherence to the 15 EBPs was lowest in Indian facilities (mean [SD], 0.42 [0.07]), whereas Pakistani (mean [SD], 0.63 [0.18]) and Indonesian (mean [SD], 0.69 [0.13]) facilities performed better. At admission (P1), birth companions were present in Indonesia (mean [SD], 0.96 [0.19]) and India (mean [SD], 1.00 [0.05]) but less so in Pakistan (mean [SD], 0.39 [0.49]). In general, adherence to practices was very low at admission in the India-based facilities, including measurement of mothers’ temperature (mean [SD], 0.00 [0.04]) and blood pressure (mean [SD], 0.03 [0.17]) as well as proper hand hygiene (mean [SD], 0.01 [0.07]), potentially driven by relatively short time windows between admission and birth.^12^ Across settings, partograph use was limited at admission (mean [SD], 0.00 [0.02] in India, 0.23 [0.42] in Indonesia, and 0.41 [0.50] in Pakistan). Shortly before birth (P2), all facilities adhered reasonably well with ensuring supplies at bedside except for the availability of the neonatal bag and mask in Indonesia (mean [SD], 0.31 [0.46]) and towels in India (mean [SD], 0.24 [0.43]). Moreover, shortly after birth (P3) in the control facilities, Indian facilities failed to administer oxytocin (mean [SD], 0.21 [0.40]). Several of the EBPs within 1 hour after birth (P4) exhibited low application rates in India; facilities in the other 2 countries performed better on P4, but neonatal temperature measurement was deficient in Indonesia (mean [SD], 0.09 [0.29]) and Pakistan (mean [SD], 0.16 [0.37]).

**Table 2.** Essential Birth Practices for the Control Group^a^

Birth practice	India		Indonesia		Pakistan	
	Observations, No.	Proportion, mean (SD)	Observations, No.	Proportion, mean (SD)	Observations, No.	Proportion, mean (SD)
Birth companion at point 1	2457	1.00 (0.05)	107	0.96 (0.19)	44	0.39 (0.49)
Maternal blood pressure taken at point 1	2457	0.03 (0.17)	108	0.94 (0.23)	44	0.82 (0.39)
Maternal temperature taken at point 1	2457	0.00 (0.04)	109	0.23 (0.42)	44	0.18 (0.39)
Partograph started at point 1	2457	0.00 (0.02)	92	0.23 (0.42)	44	0.41 (0.50)
Hand hygiene at point 1	2549	0.01 (0.07)	71	0.63 (0.49)	57	0.77 (0.42)
Clean towel available at point 2	2549	0.24 (0.43)	71	0.69 (0.47)	57	0.77 (0.42)
Clean scissor available at point 2	2549	0.87 (0.33)	70	1.00 (0.00)	56	1.00 (0.00)
Cord tie available at point 2	2549	1.00 (0.07)	70	0.97 (0.17)	57	1.00 (0.00)
Mucous extractor available at point 2	2549	0.95 (0.22)	70	0.74 (0.44)	57	0.89 (0.31)
Neonatal bag and mask at point 2	2549	0.97 (0.18)	62	0.31 (0.46)	57	0.79 (0.41)
Oxytocin administered at point 3	2547	0.21 (0.40)	70	0.96 (0.20)	57	0.98 (0.13)
Newborn weight taken at point 4	2484	0.83 (0.38)	69	0.97 (0.17)	56	0.18 (0.39)
Newborn temperature taken at point 4	2484	0.00 (0.04)	65	0.09 (0.29)	56	0.16 (0.37)
Skin-to-skin care at point 4	2484	0.08 (0.28)	66	0.70 (0.46)	56	0.38 (0.49)
Initiated breastfeeding at point 4	2484	0.04 (0.19)	66	0.53 (0.50)	56	0.21 (0.41)
Maternal temperature taken anytime	2929	0.00 (0.04)	40	0.78 (0.42)	61	0.23 (0.42)
Maternal blood pressure taken anytime	2929	0.05 (0.21)	118	1.00 (0.00)	61	0.75 (0.43)
Share of 15	2023	0.42 (0.07)	17	0.69 (0.13)	38	0.63 (0.18)

^a^ Means refer to the proportion of births in which the relevant practice was applied. Data were taken from the control groups of the respective trials, which includes births 2, 6, and 12 months after treatment initiation in India.

### SCC Outcomes

#### EBPs and Mortality

**Figure 1.**
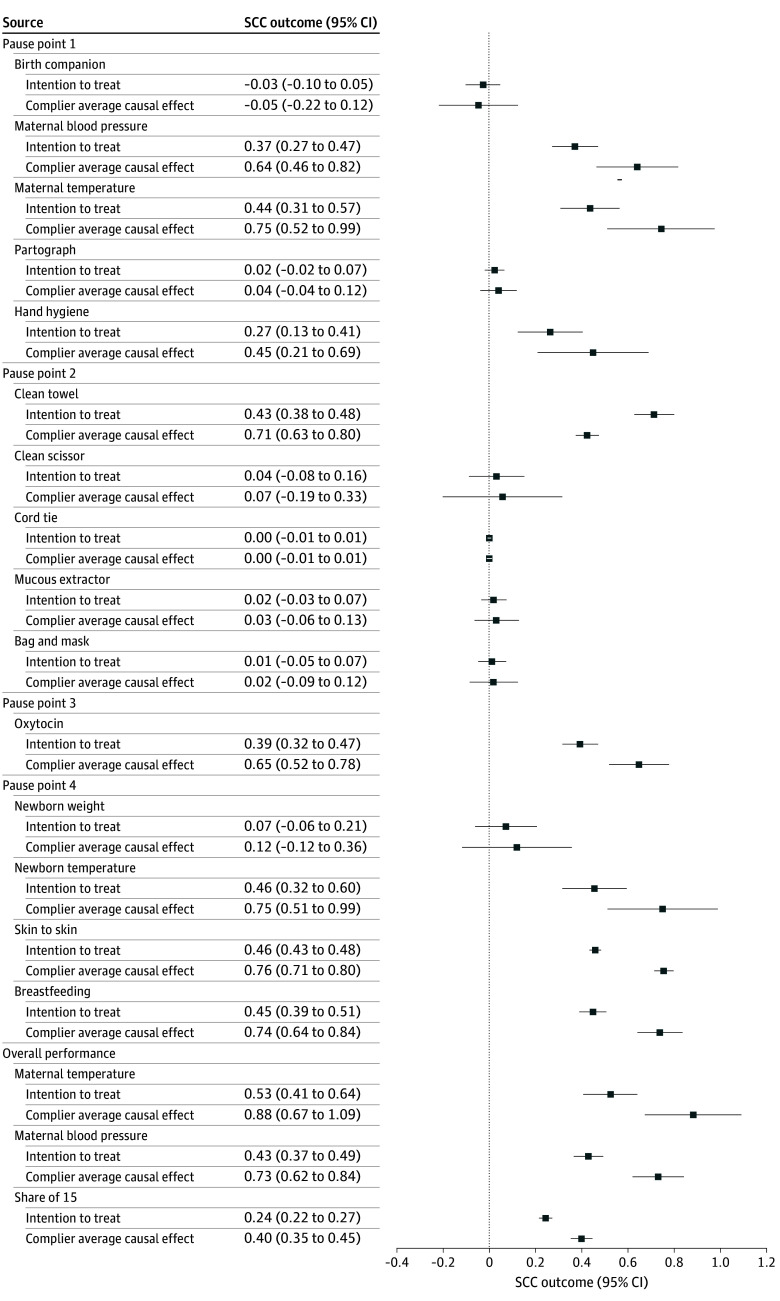
World Health Organization Safe Childbirth Checklist (SCC) and Evidence-Based Practices for the Observation Sample Error bars indicate 95% CIs. CIs may exceed 1.0 due to normal approximation.

The pooled dataset comprised 6298 observed births from the 3 cluster randomized trials. Associations between SCC and EBPs for the observation sample for both the ITT and CACE along with 95% CIs are provided in Figure 1 and eTable 3 in Supplement 1. In intervention facilities, adherence to the 15 EBPs increased by 24 percentage points (95% CI, 22 to 27; *P* < .001; q = .001; CACE) and among those who adhered by 40 percentage points (95% CI, 35 to 45; *P* < 001; q = .001; CACE). Thus, adherence to EBPs increased by 4 (ITT) to 6 (CACE) practices of the 15 EBPs. The SCC was particularly associated with increased measurement of vital signs, including neonatal temperature measurement (+46 percentage points [95% CI, 32 to 60; *P* < .001; q = .001] in the ITT analysis and +75 percentage points [95% CI, 51 to 99; *P* < .001; q = .001] in the CACE analysis) and maternal temperature (+53 percentage points [95% CI, 41 to 64; *P* < .001; q = .001] in the ITT analysis and +88 percentage points [95% CI, 67 to 109; *P* < .001; q = .001] in the CACE analysis) at any time. In contrast, newborn weight and partograph use were among the exceptions, which did not show any improvements. The partograph had a low application rate to start with, where many facilities did not have the partograph sheets and complementary supplies (eFigure 2 in Supplement 1). We did not find any associations for practices with a high level at baseline, including the supplies at bedside at P2.

**Figure 2.**
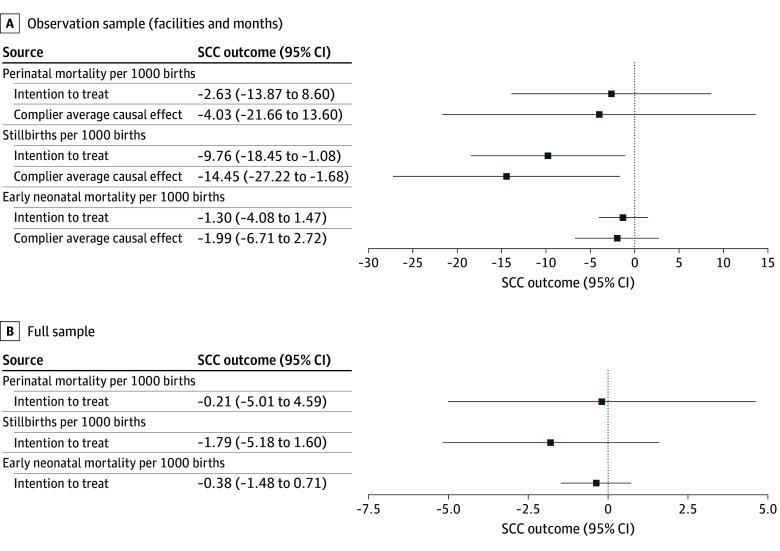
World Health Organization Safe Childbirth Checklist (SCC) and Mortality Error bars indicate 95% CIs.

Figure 2 and eTable 4 in Supplement 1 provide the ITT and CACE along with 95% CIs for the intervention associations with mortality for the full sample and the subsample of births occurring at facilities and months during which observers recorded EBP adherence. We did not detect a significant difference in mortality across the full study sample; coefficients were negative, indicating a trend toward decreasing mortality, although insignificant. However, in the subsample of intervention facilities where observations took place, the estimated stillbirth rates were lower by 9.8 per 1000 births (95% CI, −18.5 to −1.1; *P* = .03; q = .05) in the ITT analysis to 14.5 per 1000 births (95% CI, −27.2 to −1.7; *P* = .03; q = .05) in the CACE analysis **(**see eFigure 1 in Supplement 1 for analogous depiction of odds ratios). A supplementary country fixed-effects analysis (eFigure 5 in Supplement 1) demonstrated that the estimates for EBPs and mortality were largely unchanged, which reduced concerns that findings were driven by unobserved factors in a single country.

#### Heterogeneities on Primary Outcomes

We used a heterogeneity analysis to understand the potential impacts of baseline characteristics alongside the intervention on EBP adherence and mortality. Generally, the baseline characteristics, including supply availability, birth attendant experience, and facility-level maternal mortality, did not modify the outcomes (eTables 3 and 4 in Supplement 1). The heterogeneity analysis in the pooled sample suggested that the SCC was associated with increased application of EBPs across all settings, irrespective of baseline covariates (eTable 5 in Supplement 1). We also considered heterogeneous intervention associations with stillbirths as an additional outcome. The SCC was associated with a reduction of 10.7 (95% CI, −18.3 to −3.2) stillbirths per 1000 births in facilities with low stillbirth rates at baseline (*P* = .006; q = .01). The positive interaction term suggests this reduction was not observed in facilities with higher baseline stillbirth rates (*P* = .04; q = .07) (eTable 6 in Supplement 1).

#### Secondary Outcomes: Safe Childbirth Supplies and Birth Attendants’ Perceptions on Safety Culture

A complementary analysis indicated insignificant differences in the average availability of supplies between intervention and control facilities (eFigure 2 in Supplement 1). We also compared 3 questions about safety culture measures across birth attendants in all 3 trials (eFigure 3 and eTable 7 in Supplement 1). Birth attendants generally agreed that they had the necessary resources for a safe birth, but average agreement was moderate (mean [SD], 0.63 [0.15] in control facilities and 0.64 [0.15] in intervention facilities). Birth attendant confidence in performing the childbirth-related activities (mean [SD], 0.71 [0.10] in control facilities and 0.72 [0.09] in intervention facilities) and perceived safety of giving birth at their facility was scored more favorably (mean [SD], 0.84 [0.30] in control facilities and 0.86 [0.30] in intervention facilities). Although there was general agreement around perceptions of safety culture, there were no significant differences between birth attendants in the intervention vs control facilities.

## Discussion

Improving the quality of childbirth care is paramount to reducing poor maternal and neonatal health outcomes. Based on our meta-analysis, SCC use was associated with improvements in adherence to EBPs and with lower stillbirth rates in a variety of facilities. We observed stillbirth rates that were lower by 9.8 per 1000 births in facilities that received the SCC (ITT) and 14.5 per 1000 births in facilities that actively used the tool (CACE) in the subsample of facilities with on-site observations. In the full sample, estimates suggested lower stillbirth rates in facilities with low rates at baseline. There remains a missing link between behavior adherence and stillbirths, raising the question of what enabling environment factors or practices in the first 2 pause points of the SCC would drive a reduction in stillbirth because the later pause points would not influence this outcome. Detection and management of maternal high blood pressure, a sign of preeclampsia or eclampsia, or assessment of fetal heart sounds, even without use of the partograph, could have reduced the risk of stillbirth. Furthermore, the SCC induced more appropriate provision of oxytocin for labor augmentation, which may have reduced the risk of stillbirth; however, inappropriate augmentation was not measured across all 3 studies.^20^

The mechanisms by which the SCC succeeds in reducing stillbirth need to be further understood to optimize implementation across settings. In a quasi-experimental study in India and a cluster randomized study in Kenya and Uganda, locally adapted bundles of essential supplies, practitioner skills training, data quality improvements, and simulation training contributed to effective interventions.^9,21^ Given our heterogeneity analysis, providing the SCC along with coaching or monitoring was sufficient for improved practice application and outcomes in facilities with low stillbirth rates at baseline, whereas in settings with poor quality of care at baseline, more implementation support is required. Lower stillbirth rates were most evident among those who adhered, whereas estimates in the full sample did not reach statistical significance. Low implementation fidelity may have attenuated the observed associations with mortality, underscoring the need to interpret null findings with caution. Weak implementation can obscure the true potential of the SCC, and future research should focus on understanding how fidelity influences effectiveness.

### Strengths and Limitations

The strength of this meta-analysis includes harmonized data from 3 robustly designed cluster randomized trials, expanding on evidence from other studies and a systematic review, documenting significantly lower stillbirth rates (Figure 2).^9,21,22^ When considering external validity of results, we carefully accounted for potential contextual heterogeneities. A jackknife exercise that eliminates countries from the sample indicated that effects on birth practices are strongest in India (eFigure 4 in Supplement 1). Considering the implementation contexts, this finding is not surprising given that the BetterBirth trial in India had, for many practices, the lowest level at baseline and the coaching intervention was most intense in this setting. The heterogeneity in the application of practices across settings underlined previous findings that the SCC needs to be carefully adapted to the respective context to support birth attendants’ adherence.^23,24,25^ Implementers and researchers should remain aware of contextual differences across and within study settings.

Our study findings require interpretation in the context of the following limitations. Although the meta-analysis is facilitated by the similarities of the trials previously mentioned, we acknowledge differences in the country and study settings. We attempted to address this by using a country fixed-effects analysis (eFigure 5 in Supplement 1), which does not qualitatively affect the main results. Nonetheless, differences in economic and health system contexts across settings may have shaped the baseline risk environment. Variation in facility infrastructure and staffing, availability and quality of emergency obstetric and neonatal care, referral systems, and maternal sociodemographic and clinical risk profiles could have influenced the application of the checklist and mortality outcomes. Additionally, our mortality findings should be interpreted with caution given potential differences in facility type and patient characteristics across study settings. The level of complexity and risk associated with pregnancies presenting to the facilities may vary depending on the level of the health care system, which could cause bias. Our pooled sample includes predominantly primary-level facilities in India, a mix of primary and secondary hospitals in Pakistan, and a larger share of referral-level institutions in Indonesia.

Moreover, the insignificant results on birth supplies and perceptions should be considered with a grain of salt because those outcomes were not part of study protocols of all the original studies. We reported safety culture as a secondary outcome because it was measured only at the end of the study, yet it could also be understood as a mediating factor as part of the structural context that influences implementation success. In the Donabedian framework, availability of supplies and perceptions of safety culture could be considered structural elements that may moderate adherence and outcomes.^26^ Our measurement of perceptions of safety culture was based on 3 survey items, inevitably narrowing the scope compared with established instruments, such as the Agency for Healthcare Research and Quality’s Hospital Survey on Patient Safety Culture, which rely on multidimensional scales.^26,27^ Future research should use more comprehensive measures to assess the role of safety culture in SCC adherence and health outcomes.

More specific to the primary studies, measurement bias may exist due to inaccuracies in data entry in patient-level records on stillbirths and neonatal mortality. Our study may be sensitive to the Hawthorne effect on observations, particularly because no blinding of the complex intervention was possible.^28^ Although associations with EBPs remained robust even when considering the postcoaching period in all trials (eFigure 6 in Supplement 1), other research suggests that adherence decreases over time and context-tailored coaching and supervision are required to sustain behavioral change.^25,29^ Thus, further research should consider longer follow-up periods, especially to identify the drivers for sustained adherence, including general health system quality and principles of shared accountability.^30^ Another multicountry randomized clinical trial of the SCC with a context-tailored intervention and similar outcome measurement is under way in sub-Saharan Africa; thus, some of these remaining questions may be answered in the relatively near future.^31^

### Conclusions

We identified substantial associations between the SCC and increasing application of EBPs, especially at admission and after birth, as well as reduction in stillbirths when considering adherence and heterogeneity in the analysis. To realize the full potential of the SCC, implementers and policymakers need to understand better what quality infrastructure (supplies, technical skills, and shared accountability) is necessary to create an enabling environment that supports sustained SCC use.

## Notes

Supplement 1. **eTable 1. **Baseline Characteristics in Observation Sample **eTable 2.** Comparison of Baseline Characteristics Across Intervention and Control Group **eTable 3. **Treatment Effects at Endline - Essential Birth Practices **eTable 4. **Treatment Effects - Mortality at Endline **eTable 5. **Heterogeneity Analysis: Checklist Intervention Effects on the Number of Essential Birth Practices Contingent on Baseline Covariates **eTable 6. **Checklist Intervention Effects on Stillbirths Contingent on Baseline Covariates **eFigure 1. **Odds Ratios of Mortality Estimates **eFigure 2. **Supplies - Comparison of Means at Endline **eFigure 4. **Jackknife Exercise Essential Birth Practices and Mortality **eFigure 5. **EBPs and Mortality With Country-Level Fixed Effects **eFigure 6. **Treatment Effects on EBPs (Post-Coaching) **eMethods. **Supplementary Methods

Supplement 2.
Data Sharing Statement
