# Supplementary material for: The World Health Organization Safe Childbirth Checklist on Essential Birth Practices and Perinatal Mortality: A Meta-Analysis
Source: JAMA Netw Open. 2026 Feb 26;9(2):e2558269. doi: 10.1001/jamanetworkopen.2025.58269 (PMC12947022; doi:10.1001/jamanetworkopen.2025.58269)
Supplement: Supplement 1. — eTable 1. Baseline Characteristics in Observation Sample eTable 2. Comparison of Baseline Characteristics Across Intervention and Control Group eTable 3. Treatment Effects at Endline - Essential Birth Practices eTable 4. Treatment Effects - Mortality at Endline eTable 5. Heterogeneity Analysis: Checklist Intervention Effects on the Number of Essential Birth Practices Contingent on Baseline Covariates eTable 6. Checklist Intervention Effects on Stillbirths Contingent on Baseline Covariates eFigure 1. Odds Ratios of Mortality Estimates eFigure 2. Supplies - Comparison of Means at Endline eFigure 4. Jackknife Exercise Essential Birth Practices and Mortality eFigure 5. EBPs and Mortality With Country-Level Fixed Effects eFigure 6. Treatment Effects on EBPs (Post-Coaching) eMethods. Supplementary Methods [file jamanetwopen-e2558269-s001.pdf]

Supplemental Online Content

Kaplan LC, Delaney MM, Roddewig P, et al. The World Health Organization Safe Childbirth Checklist on Essential Birth Practices and Perinatal Mortality: a meta-analysis. *JAMA Netw Open*. 2026;9(2):e2558269. doi:10.1001/jamanetworkopen.2025.58269

eTable 1. Baseline Characteristics in Observation Sample
eTable 2. Comparison of Baseline Characteristics Across Intervention and Control Group
eTable 3. Treatment Effects at Endline - Essential Birth Practices
eTable 4. Treatment Effects - Mortality at Endline
eTable 5. Heterogeneity Analysis: Checklist Intervention Effects on the Number of Essential Birth Practices Contingent on Baseline Covariates
eTable 6. Checklist Intervention Effects on Stillbirths Contingent on Baseline Covariates
eTable 7. Comparison of Perceptions of Safety Culture Across Intervention and Control Group
eFigure 1. Odds Ratios of Mortality Estimates
eFigure 2. Supplies - Comparison of Means at Endline
eFigure 3. Treatment Effects on Perceptions of Safety Culture (at Endline)
eFigure 4. Jackknife Exercise Essential Birth Practices and Mortality
eFigure 5. EBPs and Mortality With Country-Level Fixed Effects
eFigure 6. Treatment Effects on EBPs (Post-Coaching)
eMethods. Supplementary Methods

This supplemental material has been provided by the authors to give readers additional information about their work.

© 2026 Kaplan LC et al. *JAMA Network Open*.

eTable 1. Baseline Characteristics in Observation Sample

	India		Indonesia		Pakistan	
	**N**	**Mean [SD]**	**N**	**Mean [SD]**	**N**	**Mean [SD]**
**Annual birth volume (n)**	30	1,888·70 [538·63]	16	698·94 [946·01]	5	1,343·20 [1,655·19]
**Perinatal death rate (per 1000)**	25	20·23 [12·04]	16	16.92 [13.71]	5	1.16 [1·68]
**Early neonatal death rate (per 1000)**	25	0·22 [0·57]	16	4·48 [7·41]	5	0·00 [0·00]
**Stillbirth rate (per 1000)**	24	20·84 [11·55]	16	12.44 [12.91]	5	1.16 [1.68]
**Maternal death rate (per 100 000)**	25	0·00 [0·00]	15	259.91 [547.18]	5	0.00 [0.00]
**Birth attendant experience (years)**	30	9·23 [4·56]	16	8.48 [4.38]	6	11.26 [5.79]
**Supply Count out of 16 supplies (n)**	30	12·07 [1·80]	16	15.19 [0.54]	5	13.40 [2.30]

Notes: Observation numbers refer to the facility level for the complier analysis (e.g., facilities in which observations took place). SD refers to standard deviations. The baseline information were not available for all variables for all facilities (India n=30, Indonesia n=17, and Pakistan n=6). Supply count refers to a facility-level count of a maximum of 16 available supplies (for a list see the e Methods in Supplement 1).

© 2026 Kaplan LC et al. *JAMA Network Open*.

eTable 2. Comparison of Baseline Characteristics Across Intervention and Control Group

Panel A: Full Sample	N<br>Treatment Facilities	Mean [SD]<br>Treatment Facilities	N<br>Control Facilities	Mean [SD]<br>Control Facilities	Difference [CI]<br>Treatment - Control Facilities
**Annual birth volume (n)**	83	1283·29<br>[845·77]	79	1321·39<br>[769·48]	-38·10<br>[-287·48 to 211·28]
**Perinatal death rate (per 1000)**	76	17·28<br>[12·04]	75	15·70<br>[15·01]	1·58<br>[-2·76 to 5·92]
**Early neonatal death rate (per 1000)**	76	0·49<br>[2·70]	75	0·65<br>[2·78]	-0·16<br>[-1·03 to 0·71]
**Stillbirth rate (per 1000)**	74	17·24<br>[12·03]	74	15·25<br>[14·80]	1·99<br>[-2·36 to 6·34]
**Maternal death rate (per 100 000)**	76	16·40<br>[120·75]	74	35·84<br>[233·97]	-19·45<br>[-78·81 to 39·91]
**Birth attendant experience (years)**	84	10·10<br>[5·58]	79	10·29<br>[5·02]	-0·18<br>[-1·82 to 1·45]
**Supply Count out of 16 supplies (n)**	83	13·02<br>[2·02]	79	12·78<br>[1·75]	0·24<br>[-0·34 to 0·82]

Panel B: Observation sample	N<br>Treatment Facilities	Mean [SD]<br>Treatment Facilities	N<br>Control Facilities	Mean [SD]<br>Control Facilities	Difference [CI]<br>Treatment - Control Facilities
**Annual birth volume (n)**	26	1,423·77<br>[1,059·34]	25	1,501·68<br>[901·68]	-77·91<br>[-618·84 to 463·02]
**Perinatal death rate (per 1000)**	23	18·98<br>[14·30]	23	15·03<br>[11·96]	3·95<br>[-3·67 to 11·57]
**Early neonatal death rate (per 1000)**	23	1·54<br>[4·80]	23	1·82<br>[4·83]	-0·28<br>[-3·06 to 2·51]
**Stillbirth rate (per 1000)**	22	18·23<br>[14·76]	23	13·21<br>[10·83]	5·02<br>[-2·52 to 12·56]
**Maternal death rate (per 100 000)**	23	54·18<br>[218·09]	22	120·57<br>[423·78]	-66·39<br>[-262·03 to 129·26]
**Birth attendant experience (years)**	27	9·35<br>[5·45]	25	9·11<br>[3·65]	0·23<br>[-2·31 to 2·78]
**Supply Count out of 16 supplies (n)**	26	13·54<br>[1·98]	25	12·80<br>[2·20]	0·74<br>[-0·41 to 1·89]

Notes: Confidence intervals (CIs) for the difference between treatment and control facilities refer to 95%. SD refers to standard deviations. The baseline information were not available for all variables for all facilities in Panel A (India n=120, Indonesia n=32, and Pakistan n=12) and in Panel B (India n=30, Indonesia n=17, and Pakistan n=6). Supply count refers to a facility-level count of a maximum of 16 available supplies (for a list see the e Methods in Supplement 1).

© 2026 Kaplan LC et al. *JAMA Network Open*.

eTable 3. Treatment Effects at Endline - Essential Birth Practices

Practice	N	ITT	ITT – CI	ITT – p-val	ITT – q-val	CACE	CACE – CI	CACE – p-val	CACE – q-val
**Birth companion at P1**	5,383	-0·03	[-0·10 to 0·05]	0·49	0·69	-0·05	[-0·22 to 0·12]	0·59	0·78
**Maternal BP taken at P1**	5,376	0·37	[0·27 to 0·47]	0·000	0·001	0·64	[0·46 to 0·82]	0·000	0·001
**Maternal temperature taken at P1**	5,372	0·44	[0·31 to 0·57]	0·000	0·001	0·75	[0·52 to 0·99]	0·000	0·001
**Partograph started at P1**	5,316	0·02	[-0·02 to 0·07]	0·29	0·49	0·04	[-0·04 to 0·12]	0·32	0·49
**Hand hygiene at P1**	5,394	0·27	[0·13 to 0·41]	0·000	0·001	0·45	[0·21 to 0·69]	0·000	0·001
**Clean towel available at P2**	5,390	0·43	[0·38 to 0·48]	0·000	0·001	0·71	[0·63 to 0·80]	0·000	0·001
**Clean scissor available at P2**	5,389	0·04	[-0·08 to 0·16]	0·50	0·70	0·07	[-0·19 to 0·33]	0·61	0·79
**Cord tie available at P2**	5,390	0·00	[-0·01 to 0·01]	0·98	1·00	0·00	[-0·01 to 0·01]	0·98	1·00
**Mucus extractor available at P2**	5,389	0·02	[-0·03 to 0·07]	0·48	0·69	0·03	[-0·06 to 0·13]	0·52	0·71
**Neonatal bag and mask at P2**	5,373	0·01	[-0·05 to 0·07]	0·70	0·85	0·02	[-0·09 to 0·12]	0·72	0·86
**Oxytocin administered at P3**	5,389	0·39	[0·32 to 0·47]	0·000	0·001	0·65	[0·52 to 0·78]	0·000	0·001
**Newborn weight taken at P4**	5,333	0·07	[-0·06 to 0·21]	0·29	0·49	0·12	[-0·12 to 0·36]	0·32	0·49
**Newborn temperature taken at P4**	5,316	0·46	[0·32 to 0·60]	0·000	0·001	0·75	[0·51 to 0·99]	0·000	0·001
**Skin-to-skin care at P4**	5,283	0·46	[0·43 to 0·48]	0·000	0·001	0·76	[0·71 to 0·80]	0·000	0·001
**Initiated breastfeeding at P4**	5,287	0·45	[0·39 to 0·51]	0·000	0·001	0·74	[0·64 to 0·84]	0·000	0·001
**Maternal temp. taken anytime**	6,117	0·53	[0·41 to 0·64]	0·000	0·001	0·88	[0·67 to 1·09]	0·000	0·001
**Maternal BP taken anytime**	6,298	0·43	[0·37 to 0·49]	0·000	0·001	0·73	[0·62 to 0·84]	0·000	0·001
**Share of 15 practices**	4,238	0·24	[0·22 to 0·27]	0·000	0·001	0·40	[0·35 to 0·45]	0·000	0·001

Notes: Results are structured along four Pause Points four pause points: at admission (P1), shortly before birth (P2), one minute after birth (P3) and within one hour after birth (P4). The analysis is based on the subset of facilities with clinical observations (India n=30, Indonesia n=16, and Pakistan n=6). Confidence intervals (CIs) refer to 95%. CIs for the CACE analysis are based on bootstrapped standard errors. Q-values adjusted for multiple hypothesis testing, following the Benjamini-Hochberg procedure.

© 2026 Kaplan LC et al. *JAMA Network Open*.

eTable 4. Treatment Effects - Mortality at Endline

**Panel A: Observation Sample (Facilities and Months)**	**N**	**ITT**	**ITT – CI**	**ITT – p-val**	**ITT – q-val**	**CACE**	**CACE – CI**	**CACE – p-val**	**CACE – q-val**
**Perinatal death rate (per 1000)**	17,453	-2·63	[-13·87 to 8·60]	0.65	0·79	-4·03	[-21·66 to 13·60]	0.65	0·79
**Stillbirth rate (per 1000)**	18,150	-9·76	[-18·45 to -1·08]	0.03	0·05	-14·45	[-27·22 to -1·68]	0.03	0·05
**Early neonatal death rate (per 1000)**	17,562	-1·30	[-4·08 to 1·47]	0.36	0·52	-1·99	[-6·71 to 2·72]	0.41	0·61

**Panel B: Observation Sample (Facilities)**	**N**	**ITT**	**ITT – CI**	**ITT – p-val**	**ITT – q-val**	**CACE**	**CACE – CI**	**CACE – p-val**	**CACE – q-val**
**Perinatal death rate (per 1000)**	53,038	1·35	[-8·98 to 11·67]	0.80	0·93	2·25	[-15·28 to 19·78]	0.80	0·93
**Stillbirth rate (per 1000)**	53,769	-3·85	[-11·36 to 3·66]	0.31	0·49	-6·33	[-18·87 to 6·21]	0.32	0·49
**Early neonatal death rate (per 1000)**	53,181	-2·41	[-6·57 to 1·74]	0.25	0·45	-4·03	[-11·56 to 3·50]	0.29	0·49

**Panel C: Full Sample**	**N**	**ITT**	**ITT – CI**	**ITT – p-val**	**ITT – q-val**	**CACE**	**CACE – CI**	**CACE – p-val**	**CACE – q-val**
**Perinatal death rate (per 1000)**	169,462	-0·21	[-5·01 to 4·59]	0.93	1·00	NA	NA	NA	NA
**Stillbirth rate (per 1000)**	169,511	-1·79	[-5·18 to 1·60]	0.30	0·49	NA	NA	NA	NA
**Early neonatal death rate (per 1000)**	168,923	-0·38	[-1·48 to 0·71]	0.49	0·69	NA	NA	NA	NA

Notes: Facilities covered refer to Panel A: (India n=30, Indonesia n=16, and Pakistan n=6). Panel B: (India n=30, Indonesia n=16, and Pakistan n=6). Panel C: (India n=120, Indonesia n=32, and Pakistan n=12). Confidence intervals (CIs) refer to 95%. CIs for the CACE analysis are based on bootstrapped standard errors. Q-values adjusted for multiple hypothesis testing, following the Benjamini-Hochberg procedure.

© 2026 Kaplan LC et al. *JAMA Network Open*.

eTable 5. Heterogeneity Analysis: Checklist Intervention Effects on the Number of Essential Birth Practices Contingent on Baseline Covariates

	N	ITT	ITT – CI	ITT – pval	ITT – q-val
**Annual Birth Volume (n)**					
Intervention	6,268	3·86	[2·80 to 4·92]	0.000	0·001
Baseline indicator (continuous)	6,268	0·07	[-0·03 to 0·18]	0.17	0·29
Baseline indicator X Intervention	6,268	-0·00	[-0·11 to 0·11]	0.950	1·00
**Birth Attendant Experience (years)**					
Intervention	6,258	4·51	[2·46 to 6·56]	0.000	0·001
Baseline indicator (continuous)	6,258	0·00	[-0·00 to 0·00]	0.57	0·78
Baseline indicator X Intervention	6,258	-0·00	[-0·00 to 0·00]	0.53	0·73
**Supply Count out of 16 supplies (n)**					
Intervention	6,258	6·34	[3·34 to 9·34]	0.000	0·001
Baseline indicator (continuous)	6,258	0·13	[-0·07 to 0·32]	0.20	0·35
Baseline indicator X Intervention (categorical)	6,258	-0·20	[-0·46 to 0·05]	0.12	0·20
**Stillbirth Rate (low=0/high=1)**					
Intervention	5,172	3·66	[2·74 to 4·58]	0.000	0·001
Baseline indicator (categorical)	5,172	-0·76	[-1·35 to -0·18]	0.01	0·02
Baseline indicator X Intervention (categorical)	5,172	0·17	[-0·97 to 1·31]	0.77	0·93
**Perinatal Mortality (low=0/high=1)**					
Intervention	5,450	3·94	[3·18 to 4·70]	0.000	0·001
Baseline indicator (categorical)	5,450	-0·76	[-1·35 to -0·18]	0.01	0·02
Baseline indicator X Intervention (categorical)	5,450	-0·23	[-1·19 to 0·72]	0.63	0·79
**Early Neonatal Mortality (low=0/high=1)**					
Intervention	5,450	3·64	[3·00 to 4·27]	0.000	0·001
Baseline indicator (categorical)	5,450	-0·06	[-0·78 to 0·66]	0.88	1·00
Baseline indicator X Intervention (categorical)	5,450	0·12	[-0·78 to 1·02]	0.80	0·93

Notes: Results are structured along four Pause Points four pause points: at admission (P1), shortly before birth (P2), one minute after birth (P3) and within one hour after birth (P4). The analysis is based on the subset of facilities with clinical observations. Confidence intervals (CIs) refer to 95%. CIs for the CACE analysis are based on bootstrapped standard errors. Q-values adjusted for multiple hypothesis testing, following the Benjamini-Hochberg procedure. We capture interactions of normally distributed variables (annual birth volume, birth attendant experience, supply count) via interactions with a continuous variable, whereas non-normally distributed variables (perinatal mortality, stillbirths, early neonatal mortality) were transformed into categories of low and high rates.

© 2026 Kaplan LC et al. *JAMA Network Open*.

eTable 6. Checklist Intervention Effects on Stillbirths Contingent on Baseline Covariates

	N	ITT	ITT – CI	ITT – p-val	ITT – q-val
**Annual Birth Volume (n)**					
Intervention	162	-8·50	[-25·79 to 8·78]	0.34	0·51
Baseline indicator (continuous)	162	-0·00	[-0·01 to 0·01]	0.92	1·00
Baseline indicator X Intervention	162	0·00	[-0·01 to 0·01]	0.47	0·69
**Birth Attendant Experience (years)**					
Intervention	163	-2·11	[-11·09 to 6·87]	0.65	0·79
Baseline indicator (continuous)	163	-0·23	[-0·80 to 0·33]	0.42	0·61
Baseline indicator X Intervention	163	-0·19	[-0·90 to 0·51]	0.59	0·78
**Supply Count out of 16 supplies (n)**					
Intervention	162	-6·89	[-45·71 to 31·94]	0.73	0·86
Baseline indicator (continuous)	162	-0·53	[-3·38 to 2·33]	0.72	0·86
Baseline indicator X Intervention (categorical)	162	0·24	[-3·03 to 3·52]	0.88	1·00
**Stillbirth Rate (low=0/high=1)**					
Intervention	148	-10·73	[-18·31 to -3·15]	0.006	0·01
Baseline indicator (categorical)	148	3·50	[-4·81 to 11·82]	0.41	0·61
Baseline indicator X Intervention (categorical)	148	11·04	[0·62 to 21·46]	0.04	0·07
**Perinatal Mortality (low=0/high=1)**					
Intervention	151	-9·46	[-17·03 to -1·89]	0.02	0·03
Baseline indicator (categorical)	151	3·99	[-4·28 to 12·27]	0.35	0·51
Baseline indicator X Intervention (categorical)	151	9·01	[-1·37 to 19·39]	0.09	0·15
**Early Neonatal Mortality (low=0/high=1)**					
Intervention	151	-4·20	[-10·52 to 2·11]	0.19	0·34
Baseline indicator (categorical)	151	-0·53	[-9·67 to 8·61]	0.91	1·00
Baseline indicator X Intervention (categorical)	151	0·94	[-12·22 to 14·09]	0.89	1·00

Notes: Confidence intervals refer to 95%. Q-values adjusted for multiple hypothesis testing, following the Benjamini-Hochberg procedure. We capture interactions of normally distributed variables (annual birth volume, birth attendant experience, supply count) via interactions with a continuous variable, whereas non-normally distributed variables (perinatal mortality, stillbirths, early neonatal mortality) were transformed into categories of low and high rates.

© 2026 Kaplan LC et al. *JAMA Network Open*.

eTable 7. Comparison of Perceptions of Safety Culture Across Intervention and Control Group

	N<br>Treatment Facilities	Mean [SD]<br>Treatment Facilities	N<br>Control Facilities	Mean [SD]<br>Control Facilities	Difference [CI]<br>Treatment - Control Facilities
**Save to have baby at facility**	1,156	0·86<br>[0·30]	1,199	0·84<br>[0·30]	0·01<br>[-0·04 to 0·07]
**Necessary resources for care**	1,353	0·64<br>[0·15]	1,353	0·63<br>[0·15]	0·003<br>[-0·02 to 0·03]
**Confidence in activities**	1,163	0·72<br>[0·09]	1,352	0·71<br>[0·10]	0·009<br>[-0·002 to 0·02]

Notes: Confidence intervals (CIs) for the difference between treatment and control facilities refer to 95%. SD refers to standard deviations.

© 2026 Kaplan LC et al. *JAMA Network Open*.

eFigure 1. Odds Ratios of Mortality Estimates

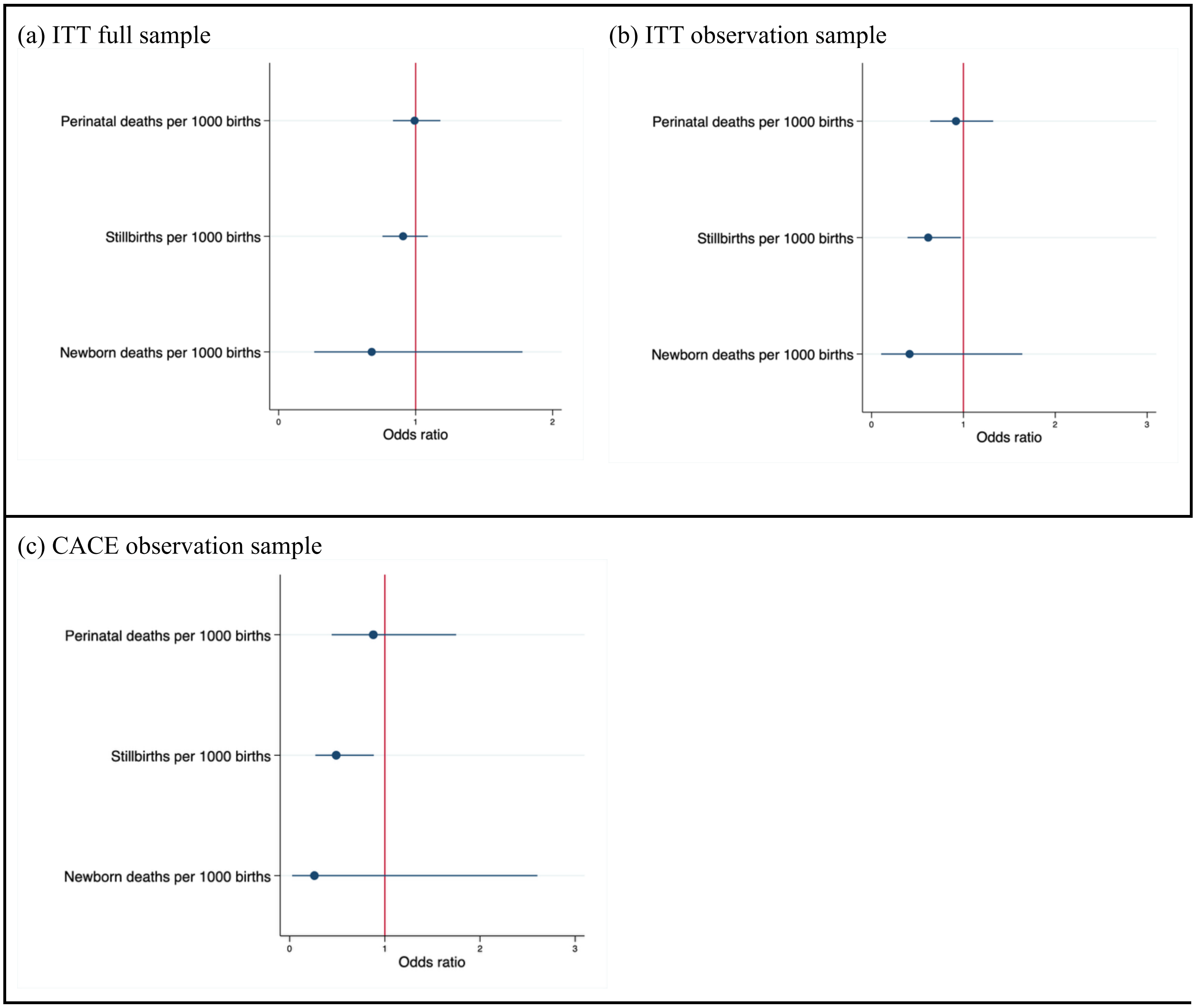

Notes: Confidence intervals (CIs) refer to 95%. Results from a Generalized Linear Model link(logit); binomial distribution. CIs for the CACE analysis are based on bootstrapped standard errors. The observation sample constrains the analysis to facilities and months in which observations were conducted.

© 2026 Kaplan LC et al. *JAMA Network Open*.

eFigure 2. Supplies - Comparison of Means at Endline

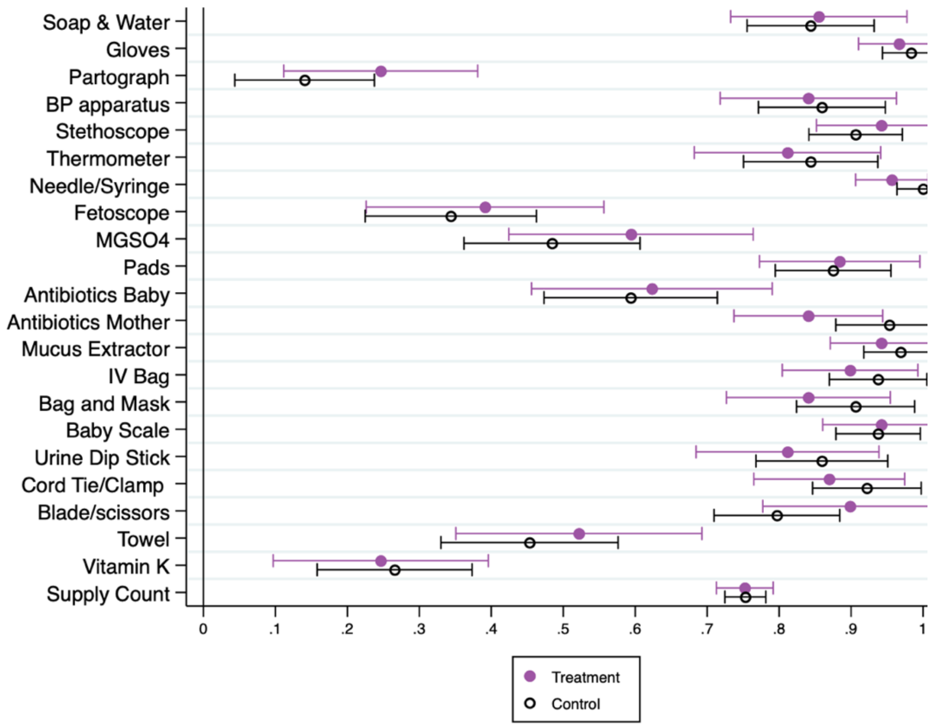

Notes: Figure depicts means of proportions across facilities in intervention and control group at endline. Confidence intervals (CIs) refer to 95%. CIs may exceed 1.0 due to normal approximation.

© 2026 Kaplan LC et al. *JAMA Network Open*.

eFigure 3. Treatment Effects on Perceptions of Safety Culture (at Endline)

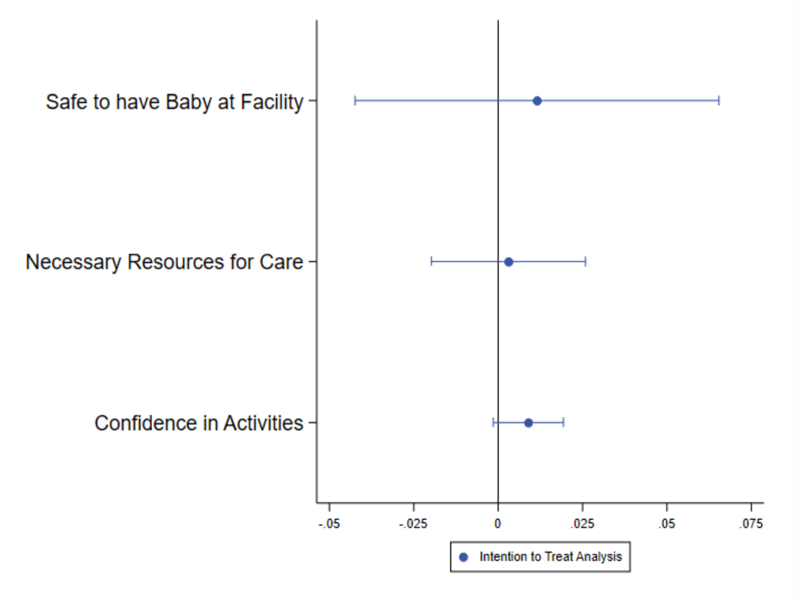

Notes: Results from a Generalized Linear Model link(logit); binomial distribution. Confidence intervals refer to 95%.

© 2026 Kaplan LC et al. *JAMA Network Open*.

eFigure 4. Jackknife Exercise Essential Birth Practices and Mortality

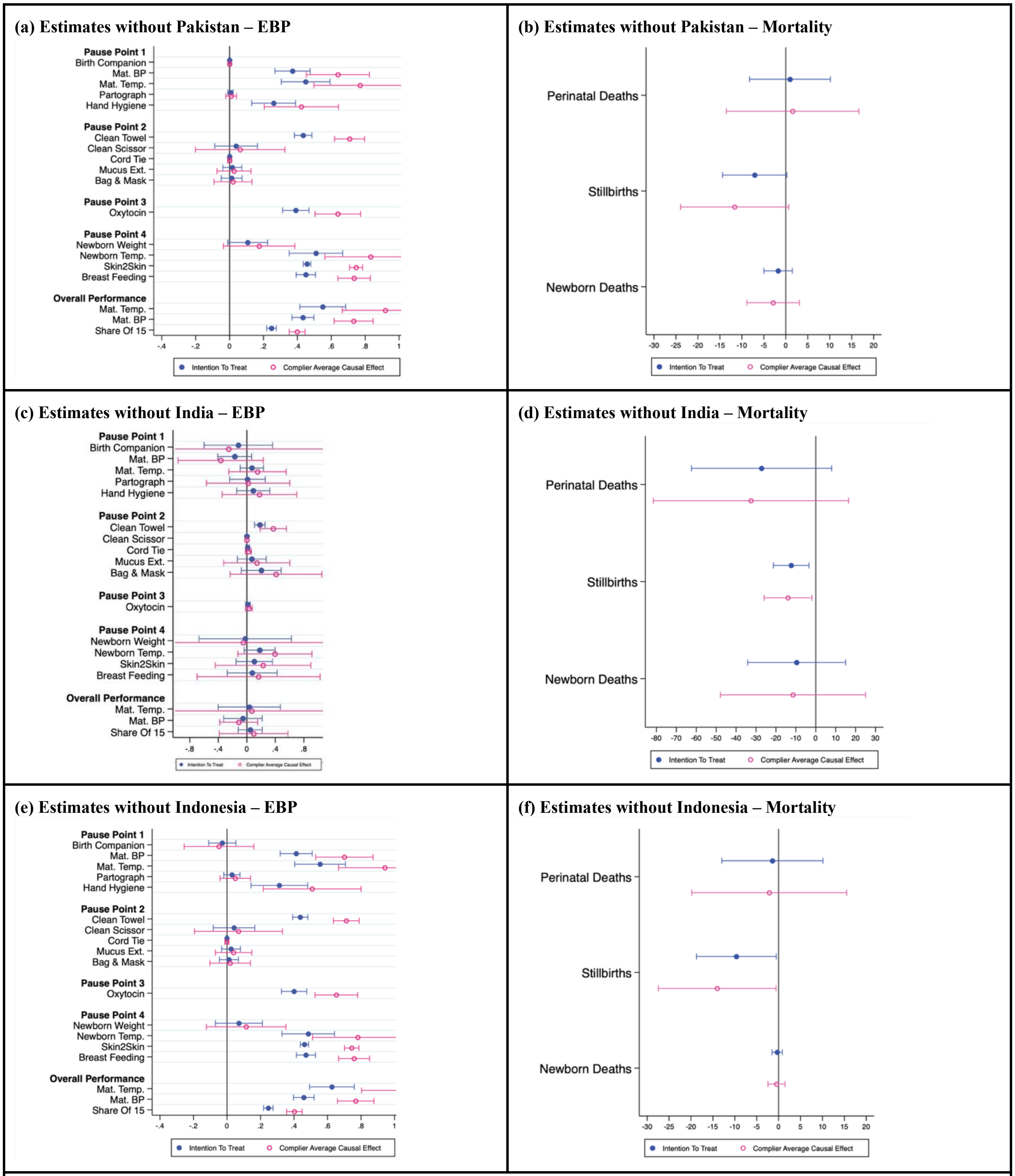

Notes: Results are from a jackknife exercise, which drops each country one by one. Confidence intervals (CIs) refer to 95%. CIs for the CACE analysis are based on bootstrapped standard errors. CIs may exceed 1.0 due to normal approximation.

© 2026 Kaplan LC et al. *JAMA Network Open*.

eFigure 5. EBPs and Mortality With Country-Level Fixed Effects

(a) Essential Birth Practices

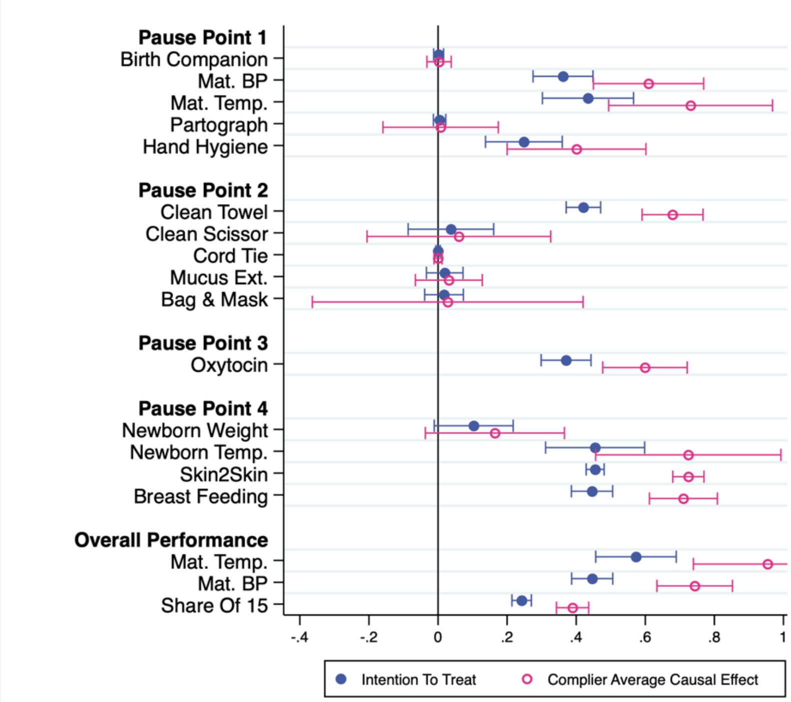

(b) Mortality

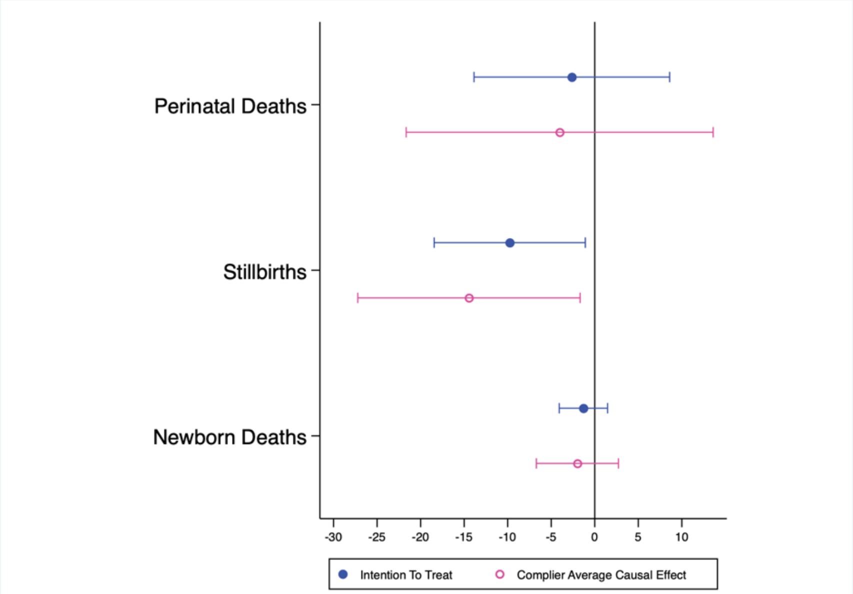

Notes: Figures are analogous to Figure 1 and Figure 2(b) but considering country-level fixed effects as an additional covariate. Confidence intervals (CIs) refer to 95%. CIs for the CACE analysis are based on bootstrapped standard errors. CIs may exceed 1.0 due to normal approximation.

© 2026 Kaplan LC et al. *JAMA Network Open*.

eFigure 6. Treatment Effects on EBPs (Post-Coaching)

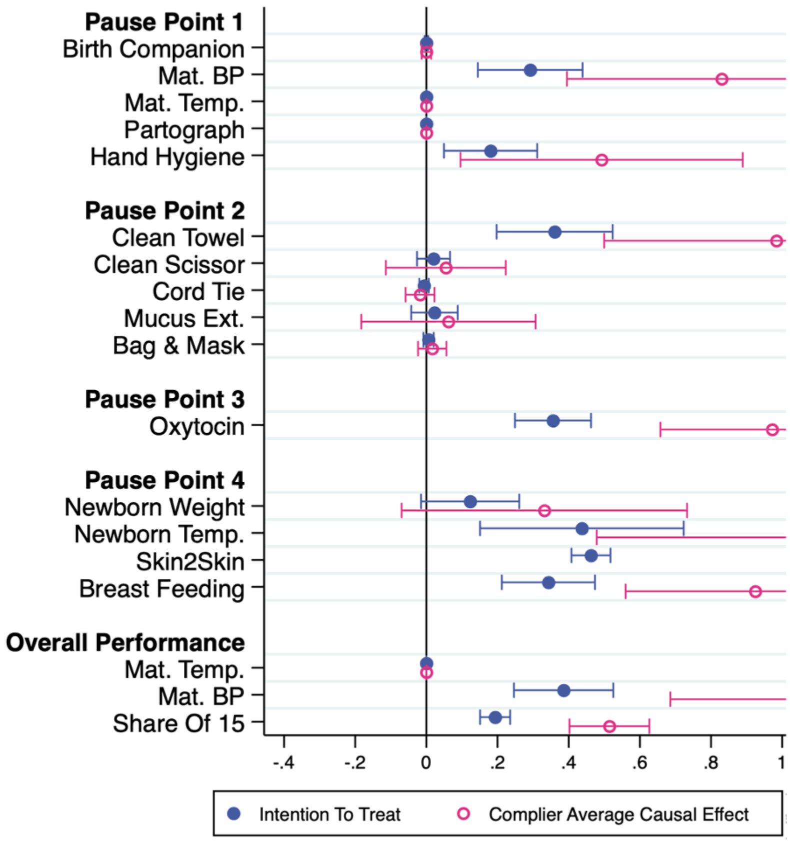

Notes: This figure presents point estimates analogous to Figure 1, but excluding observations conducted in India during the ongoing coaching phase. Coefficients can thus be interpreted as lower bound estimates. Confidence intervals (CIs) refer to 95%. CIs for the CACE analysis are based on bootstrapped standard errors. CIs may exceed 1.0 due to normal approximation.

© 2026 Kaplan LC et al. *JAMA Network Open*.

eMethods. Supplementary Methods

Essential Birth Practices

<u>**Practice**</u>	<u>**Description**</u>	<u>**Practice**</u>	<u>**Description**</u>
**Birth Companion**	Birth companion present at P1 (one of the 15 EBPs)	**Bag & Mask**	Neonatal bag and mask available at P2 (one of the 15 EBPs)
**Mat. BP**	Maternal blood pressure taken at P1	**Oxytocin**	Oxytocin administered at P3 (one of the 15 EBPs)
**Mat. Temp.**	Maternal temperature taken at P1	**Newborn Weight**	Newborn weight taken at P4 (one of the 15 EBPs)
**Partograph**	Partography started at P1 (one of the 15 EBPs)	**Newborn Temp.**	Newborn temperature taken at P4 (one of the 15 EBPs)
**Hand Hygiene**	Hand hygiene at P2 (one of the 15 EBPs)	**Skin2Skin**	Skin-to-skin care initiated at birth at P4 (one of the 15 EBPs)
**Clean Towel**	Clean towel available at P2 (one of the 15 EBPs)	**Breast Feeding**	Initiation of breastfeeding at P4 (one of the 15 EBPs)
**Clean Scissor**	Clean scissors or blade available at P2 (one of the 15 EBPs)	**Mat. Temp.**	Maternal temperature taken anytime (one of the 15 EBPs)
**Cord Tie**	Cord tie available at P2 (one of the 15 EBPs)	**Mat. BP**	Maternal blood pressure taken anytime (one of the 15 EBPs)
**Mucus Ext.**	Mucus extractor available at P2 (one of the 15 EBPs)	**Share Of 15**	Share of the 15 EBPs considered

Notes: The table describes the EBPs considered as outcomes in this study. We considered 15 EBPs (out of 18 essential birth practice) identified in Semrau et al. (2017), for which we could harmonize data.

© 2026 Kaplan LC et al. *JAMA Network Open*.

Supplies at baseline

For the baseline analysis, we considered the following 16 essential birth supplies that were available across the three trials, namely:

Gloves; partograph sheets; Blood Pressure Apparatus; Stethoscope; Thermometer; Needles and Syringe; Fetoscope; Magnesium Sulphate; Mucus Extractor; IV and Bag; Bag and Mask; Baby Scale; Cord Tie/Clamp; Blade/Scissor; Towel; Soap and Water (or alcoholic handrub)

Safety culture

We used three safety culture indicators, which were collected in a similar way across settings, but with wording adjusted for the specific context. Those items more specifically read as:

Country/Measure	Safe to have baby at facility	Necessary resources for care	Confidence in activities
**India**	Would you feel safe having your baby in this health facility?	During the last 6 months, how often did you have the necessary resources to deliver safe care for mothers and babies?<br>Necessary resources include medicines,<br>equipment and supplies.	During the last 6 months, how often did you feel confident that you knew which activities you needed to do at every childbirth?
**Indonesia**	I would feel safe being treated here as a delivery patient.	Do you have access to the tools and resources to do your job well?	If all supplies and equipment would be available in your facility, how secure would you feel that your team can administer a safe delivery?
**Pakistan**	I would feel safe being treated here as a delivery patient.	Do you have access to the tools and supplies to do your job well?	If all supplies and equipment would be available, how secure would you feel that you can administer a safe delivery?

© 2026 Kaplan LC et al. *JAMA Network Open*.

Study screening

Screening procedure
We conducted a systematic literature search on Google Scholar for the term "Safe Childbirth Checklist" covering the years 2009 (design phase of the Checklist) until 2021. Above English language sources, we also considered Portuguese and Spanish since we were aware of different implementation projects in South America. Based on the literature review and the implementation network of Ariadne Labs, we identified 35 implementation sides (see table below).

Africa	Americas	Asia	Eastern Mediterranean	Europe
Ethiopia<br>Guinea<br>Mali<br>Namibia<br>Nigeria<br>Tanzania<br>Uganda	Argentina<br>Brazil<br>Colombia<br>Mexico<br>Peru<br>Uruguay<br>Venezuela	Bangladesh<br>China<br>India<br>Indonesia<br>Philippines<br>Sri Lanka	Egypt<br>Iran<br>Lebanon<br>Pakistan<br>Sudan	Italy<br>Spain

Inclusion criteria and trial descriptions
We consider data from the three existing randomized trials, which evaluate the Checklist against a standard of care control group without use of the tool. The table below provides an overview of the study characteristics. For data from Pakistan (Kuhnt, Hashmi and Vollmer, 2023), we exclude the community midwives and lady health visitors and focus on the 12 health facilities included to ensure comparability across samples.

	Population	Study Type	Dates	Intervention	Participants	Controls
**Semrau et al. (2017)**	120 primary and community health centers	RCT	2014 to 2016	Checklist plus 43 coaching visits	60	60
**Kaplan et al. (2021)**	32 primary and secondary level health facilities (at least BEmONC)	RCT	2016 to 2017	Checklist plus 11 coaching visits	16	16
**Kuhnt, Hashmi and Vollmer (2023)**	108 Community midwives, lady health visitors as well as 12 primary and secondary level health facilities	RCT	2016 to 2017	Checklist plus monthly monitoring visits	61	59

© 2026 Kaplan LC et al. *JAMA Network Open*.

Components of included studies impacting pooling of data

In the following, we briefly describe the key components of each trial that impacted the pooling of data.

India: The BetterBirth Trial was a matched-pair, cluster-randomized controlled trial in 60 intervention facilities and 60 control facilities in Uttar Pradesh, India. Participating primary and community health centers had at least 1,000 birthing processes annually; at least three birth attendants with training of at least the level of an auxiliary nurse midwife; no other concurrent quality-improvement or research programs; and district and facility leadership willing to participate (ClinicalTrials.gov NCT02148952).[1,2] The initial study's sample size was determined to capture hypothesized treatment effects for EBPs and mortality outcomes.[1] Facilities (i.e., clusters) were matched based on geographic zone, facility type, distance to a district hospital, annual birth volume, and number of birth attendants, and then randomly assigned to treatment within each matched pair. The 8-month long intervention involved 43 day-long coaching visits, starting twice weekly in the first four months, to weekly to monthly in the second four months.[3,4] Baseline data on birth volume and supply availability were collected before the start of the intervention, while data on baseline mortality and birth attendants' years of experience were collected retrospectively. Health outcomes data collection began two months after intervention initiation, continuing for up to 12 months. EBPs were directly observed at approximately 2, 6, and 12 months after initiation. Data collection spanned November 2014 to January 2017.

Indonesia: The "Safe Childbirth in Indonesia" study (ISRCTN11041580) was a matched-group, cluster-randomized controlled trial in 16 intervention and 16 control facilities in three districts of Aceh Province. Facilities provided at minimum Basic Emergency Obstetric and Newborn Care.[5] Facilities were matched into two groups based on an optimization approach to balance facility characteristics across trial arms, after which treatment was randomly assigned. The initial study's sample size was determined to capture hypothesized treatment effects for EBPs, but not for mortality outcomes.[5] Treatment included 11 two-hour long coaching visits over six months with three visits in the first month, reduced to one visit per month in the last month. Baseline data was collected before implementation in September/October 2016, and endline data in March/April 2017, one month after coaching ended. Data on deaths and births was transformed into patient-level information based on case numbers from facility registries.

Pakistan: The matched-pair, cluster-randomized controlled trial covered two districts of Khyber Pakhtunkhwa Province in the northwest of Pakistan. The original study evaluated 2,839 births across 12 primary (basic and rural health centers) and secondary level (hospitals) health facilities as well as 1,287 births from 108 individual health workers conducting primarily home-based births.[6] Providers were grouped into strata defined by health cadre and district and then matched in pairs within strata, where treatment was randomly assigned. Matching variables included the provider's total number of deliveries as well as their mortality, referral, and birth complication rates. For comparability, our analysis focuses on 12 facilities consistent with the SCC's facility-based design. Eligible facilities had at least four births per month. The original study's sample size was determined to capture hypothesized treatment effects for EBPs, but not for mortality outcomes.[6] Implementation included monthly light-touch monitoring, a four-day skills training for both study arms, and supply assessments with support provided equally to intervention and control facilities, addressed by the Deutsche Gesellschaft für Internationale Zusammenarbeit (GIZ), making it the least intensive intervention across the three settings. Data were collected from November 2016 until July 2017. Observers documented EBPs two months post intervention. Since the intervention was phased-in across facilities, we consider facility-level information on mortality for nine months of full treatment.

© 2026 Kaplan LC et al. *JAMA Network Open*.

Flow Diagram for Randomization and Data Collection

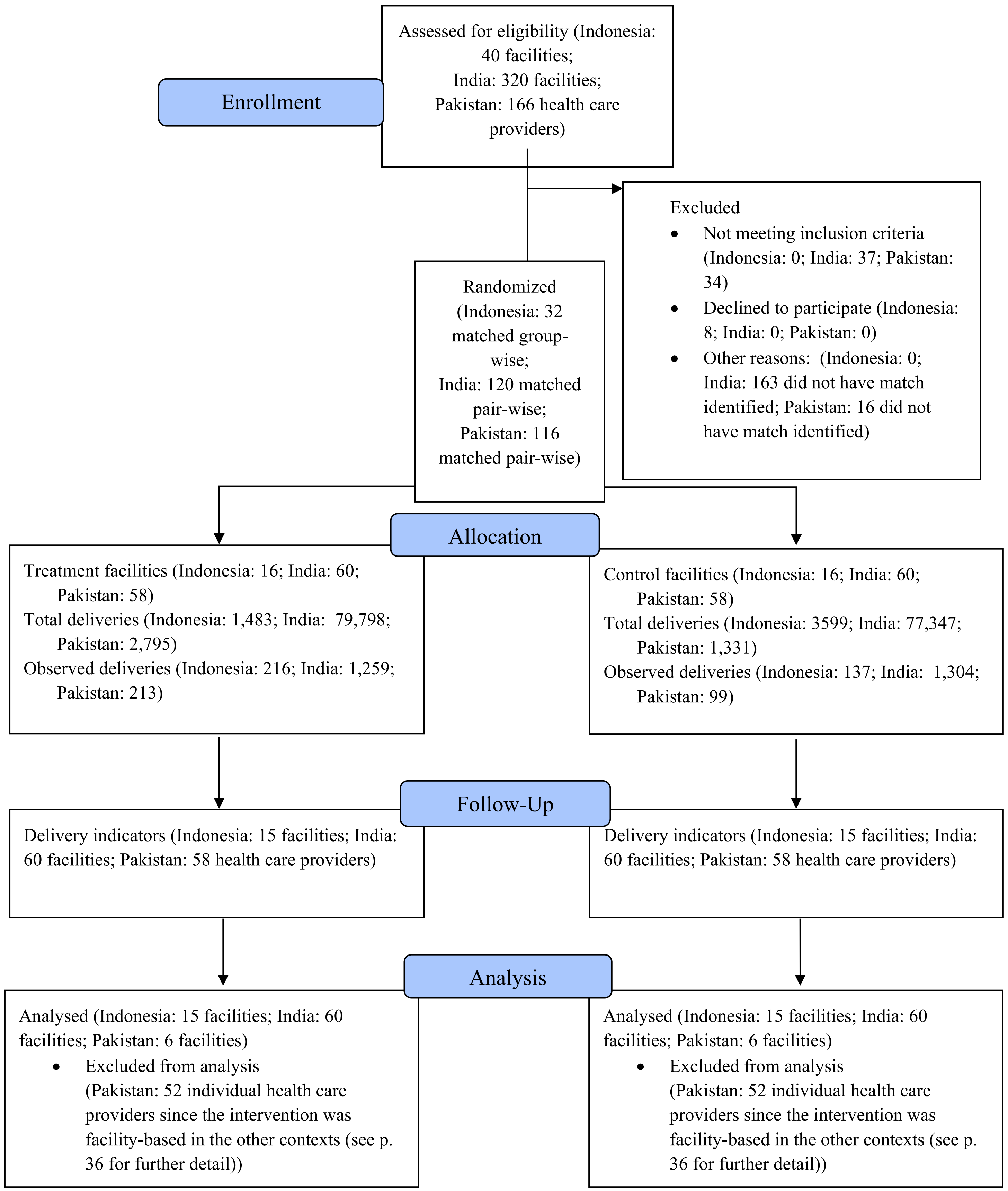

© 2026 Kaplan LC et al. *JAMA Network Open*.

Additional statistical details

Intention To Treat Analysis

For the ITT analysis, we estimate following parsimonious model:

$$Y_{ij} = \alpha + \beta_1 * T_{ij} + \varepsilon_{ij}$$

where Y_{ij} refers to outcomes at the birth (essential practices for birth i at facility j) or facility level (health outcomes at facility j), which are regressed on the treatment status T_{ij}.

Complier Average Causal Effect (CACE) Analysis

The CACE approach builds on a two-step analysis, where the treatment status T_j at facility j serves as an instrument to predict compliance in the first stage:

$$C_{ij} = \alpha + \beta_1 * T_j + \varepsilon_{ij}$$

Where C_{ij} indicates compliance (Checklist use) at birthing process i at facility j. Predicted compliance is considered as an explanatory variable in the 2nd stage to estimate analogous to the ITT regression:

$$Y_{ij} = \alpha + \beta_1 * \hat{C}_{ij} + \varepsilon_{ij}$$

where Y_{ij} refers to outcomes at the birth (essential practices for birth i at facility j) or facility level (health outcomes at facility j), which are regressed on predicted compliance. Compliance is measured at the birth level by midwives actively using or looking at the Checklist during clinical observations. When analyzing facility-level outcomes with a CACE, we measured compliance by calculating the number of observed births with Checklist use over the total numbers of observed births per facility: Compliance=Observed births with Checklist use/total observed births

While individuals from the control group may also theoretically qualify as compliers if they would use the Checklist (also labeled "always-takers" in the literature), this case did not materialize during observed births. Our clustered trial design (provision of Safe Childbirth Checklist at facility-level) made spill-overs very unlikely to happen.

Heterogeneity

In order to assess heterogeneities, we estimate a model analogous to our ITT analysis via an Ordinary Least Squares Model which adds an interaction of the Checklist intervention with facility-level baseline characteristics.

For the number of EBPs the estimation equation looks like:

$$EBP_i = \alpha + \beta_1 * treat_i * X_i + \beta_2 * treat_i + \beta_3 * X_i + \varepsilon_i$$

where i is a specific birth observation, treat refers to treatment status and X_i to the covariate of interest.

For endline stillbirth rates (measured in x/1000 at the facility level) the estimation equation looks like:

$$Stillbirths_i = \alpha + \beta_1 * treat_i i * X_i + \beta_2 * treat_i + \beta_3 * X_i + \xi_i$$

where i is the facility of interest, treat refers to treatment status and X_i to the covariate of interest.

© 2026 Kaplan LC et al. *JAMA Network Open*.

We capture interactions of normally distributed baseline covariates (annual birth volume, birth attendant experience, supply count) via a continuous variable, whereas non-normally distributed baseline covariates (perinatal mortality, stillbirths, early neonatal mortality) were transformed into categories of low and high rates.

Research team 'reflexivity' statements

In alignment with the recent discussion around equitable authorship and collaboration, we have prepared a structured reflexivity statement based on the recent article by Morton *et al.* (2021)[7]

1. **How does this study address local research and policy priorities?**
   Maternal and newborn mortality remain too high; particularly in resource-constrained settings. Alongside systems-level interventions, facility-level interventions are needed to reduce mortality and poor quality of care. The WHO Safe Childbirth Checklist is a potentially effective tool to address quality of care and health outcomes. This pooled analysis builds on three randomized controlled trials that were already completed in India, Pakistan, and Indonesia.

2. **How were local researchers involved in study design?**
   Local researchers in India, Pakistan, and Indonesia were co-principal investigators on the original studies. For the Indonesia study, the intervention (treatment) and questionnaires were designed by Ichsan, Marthoenis, Farah Diba, and Muhsin in Indonesia and Ashfa Hashmi in Pakistan. For the BetterBirth trial, Dr. Vishwajeet Kumar and his team at Community Empowerment Lab co-designed the BetterBirth trial including study protocol, data collection tools, and data interpretation. For our pooled analysis, the local leadership and research teams are co-authors on the paper, and as such reviewed the analysis and paper. All three studies worked in collaboration with the international teams.

3. **How has funding been used to support the local research team(s)?**
   For this pooled study, the only funding available was for Dr. Lennart Kaplan who received an award from the German Academic Exchange Service for a post-doctoral fellowship at Ariadne Labs. All other authors in high and low resource settings did not receive funding for this work.

4. **How are research staff who conducted data collection acknowledged?**
   All research staff and enumerators from the original trials are acknowledged in the Acknowledgement section of this paper for their leadership in collection of the original trials. For the pooled analysis, the local trial leaders are included as co-authors on this manuscript.
5. **How have members of the research partnership been provided with access to study data?**
   For this pooled analysis, the de-identified dataset will be available to the co-authors and the full dataset will be made available to the public. For the individual trials, the availability of the datasets varied with PI groups.
6. **How were data used to develop analytical skills within the partnership?**
   In the pooled analysis, Dr. Lennart Kaplan led the analysis and worked on the data interpretation with co-authors through email communication and phone calls. There was not an intentional process to improve analytical skills across the partnership.

7. **How have research partners collaborated in interpreting study data?**
   Through a collaborative document, we shared results and co-authors commented on the manuscript. We organized joint calls to discuss final findings with the co-authors/partners and incorporate qualitative findings in the interpretation of results. All co-authors provided feedback on the final manuscript.

© 2026 Kaplan LC et al. *JAMA Network Open*.

8. **How were research partners supported to develop writing skills?**
   Through a collaborative document, we shared results and co-authors commented on the manuscript. We organized joint calls to discuss final findings with the co-authors/partners and incorporate qualitative findings in the interpretation of results.
9. **How will research products be shared to address local needs?**
   The study results from the original trials were shared through dissemination efforts locally and globally. For this pooled analysis, the full co-author list will be able to share in-country with key stakeholders. Further, the results will be shared with the WHO-HQ, WHO country offices, and the World Bank.

10. **How is the leadership, contribution and ownership of this work by LMIC researchers recognised within the authorship?**
    The individual trials each had papers with LMIC researchers as first authors (e.g., Diba et al. 2019 and Kaplan et al. 2021 in Indonesia; joint first authorship of *JAMA Open* article in Indonesia; co-senior authorship for the NEJM BetterBirth trial). For this article, contribution and leadership of the studies is recognized through co-authorship (50% LMIC; 50% HIC).
11. **How have early career researchers across the partnership been included within the authorship team?**
    Dr. Lennart Kaplan, a non-tenured researcher, is the first author of this manuscript. The majority of the authors are early or mid-career professionals from India, Indonesia, Pakistan, Germany & the United States. Drs. Kumar, Hizir, Semrau, and Vollmer are more advanced in their careers and provided mentorship for this work. Further, each trial also encouraged non-tenured scholars as first authors (e.g., Diba et al. 2019; Doria et al. 2019).

12. **How has gender balance been addressed within the authorship?**
    Of the 18 authors, 10 identify as female; thus we have achieved near gender balance of the authorship.

13. **How has the project contributed to training of LMIC researchers?**
    In this pooled analysis, we did not have additional training of LMIC researchers other than co-authorship involved data interpretation, writing and messaging.

14. **How has the project contributed to improvements in local infrastructure?**
    This pooled analysis has not directly contributed to improvements in local infrastructure.

15. **What safeguarding procedures were used to protect local study participants and researchers?**
    There was no primary data collection as part of this pooled analysis, therefore, this question is not directly applicable. However, we do want to note that local study participants and researchers were part of the original study teams and dissemination of study results. Ethical review and community participation were part of the study establishment and dissemination of results.

© 2026 Kaplan LC et al. *JAMA Network Open*.

eReferences

1. Semrau KEA, Hirschhorn LR, Marx Delaney M, et al. Outcomes of a Coaching-Based WHO Safe Childbirth Checklist Program in India. *N Engl J Med*. 2017;377(24):2313–2324.

2. Semrau KEA, Hirschhorn LR, Kodkany B, et al. Effectiveness of the WHO Safe Childbirth Checklist program in reducing severe maternal, fetal, and newborn harm in Uttar Pradesh, India: study protocol for a matched-pair, cluster-randomized controlled trial. *Trials.* 2016;17(1):576.

3. Kara N, Firestone R, Kalita T, et al. The BetterBirth Program: Pursuing Effective Adoption and Sustained Use of the WHO Safe Childbirth Checklist Through Coaching-Based Implementation in Uttar Pradesh, India. *Glob Health Sci Pract*. 2017;5(2):232–243.

4. Marx Delaney M, Maji P, Kalita T, et al. Improving adherence to essential birth practices using the WHO safe childbirth checklist with peer coaching: experience from 60 public health facilities in Uttar Pradesh, India. *Glob Health Sci Pract*. 2017;5(2): 217–231.

5. Kaplan LC, Ichsan I, Diba F, et al. Effects of the World Health Organization Safe Childbirth Checklist on Quality of Care and Birth Outcomes in Aceh, Indonesia: A Cluster-Randomized Clinical Trial. *JAMA Netw Open.* 2021;4(12):e2137168.

6. Kuhnt J, Hashmi, A, Vollmer S. The effect of the WHO safe childbirth checklist on essential delivery practices and birth outcomes: Evidence from a pair-wise matched randomized controlled trial in Pakistan. *SSM-Population Health*. 2023;24:101495.

7. Morton B, Vercueil A, Masekela R, et al. Consensus statement on measures to promote equitable authorship in the publication of research from international partnerships. *Anaesthesia.* 2021;77(3): 264-276.

© 2026 Kaplan LC et al. *JAMA Network Open*.
