# Supplementary material for: The World Health Organization Safe Childbirth Checklist on Essential Birth Practices and Perinatal Mortality: A Meta-Analysis
Source: JAMA Netw Open. 2026 Feb 26;9(2):e2558269. doi: 10.1001/jamanetworkopen.2025.58269 (PMC12947022; doi:10.1001/jamanetworkopen.2025.58269)
Supplement: Supplement 2. — Data Sharing Statement [file jamanetwopen-e2558269-s002.pdf]

Data Sharing Statement

Kaplan. The World Health Organization Safe Childbirth Checklist on Essential Birth Practices and Perinatal Mortality. *JAMA Netw Open*. Published February 26, 2026. doi:10.1001/jamanetworkopen.2025.58269

Data
Data available: Yes
Data types: Data dictionary
How to access data: The data are available upon request by e-mailing to svollmer@uni-goettingen.de. We plan to publish the data.
When available: With publication

Supporting Documents
Document types: None

Additional Information
Who can access the data: Anyone requesting the data
Types of analyses: Replication
Mechanisms of data availability: Signed data access agreement
